# 2,5-dimethyl celecoxib induces apoptosis and autophagy via activation of ROS/JNK axis in nasopharyngeal carcinoma cells

**DOI:** 10.18632/aging.203488

**Published:** 2021-09-12

**Authors:** Tan Tan, Xiangjie Fu, Jiaquan Qu, Miao Zhang, He Chen, Yaochun Wang, Bo Wang, Juan Li, Jie Liu, Peijun Liu

**Affiliations:** 1 Center for Translational Medicine, The First Affiliated Hospital of Xi’an Jiaotong University, Shanxi 710061, China; 2 Center for Precision Medicine, Affiliated to The First People’s Hospital of Chenzhou, University of South China, Chenzhou 423000, China; 3 Department of Blood Transfusion, Clinical Transfusion Research Center, Xiangya Hospital, Central South University, Changsha 410008, China; 4 Cholestatic Liver Diseases Center and Department of Gastroenterology, Southwest Hospital, Third Military Medical University, Chongqing 400038, China

**Keywords:** 2,5-dimethyl celecoxib, autophagy, apoptosis, nasopharyngeal carcinoma

## Abstract

2,5-dimethyl celecoxib (DMC), a close derivative of celecoxib, has also been reported to have anticancer effects. However, the effects and underlying molecular mechanisms of DMC with respect to nasopharyngeal carcinoma are still largely unknown. In this study, we present that DMC has displayed anticancer potency in nasopharyngeal carcinoma *in vitro* and *in vivo*. Mechanistically, we found DMC induced apoptosis and autophagy for anticancer therapy against nasopharyngeal carcinoma. Furthermore, DMC-induced autophagy could remarkably attenuate after the treatment of reactive oxygen species (ROS) scavenger N-acetyl cysteine (NAC) and c-Jun N-terminal kinase (JNK) inhibitor SP600125 (SP). Taken together, these results suggested DMC induced apoptosis and autophagic death via activation of ROS/JNK axis in NPC cells, which providing us new insights into developing potential therapeutic agents for nasopharyngeal carcinoma patients.

## INTRODUCTION

Nasopharyngeal carcinoma (NPC) is a malignancy arising from nasopharyngeal epithelial cells. The incidence of NPC is high in Southern China, especially the Guangdong province and the Northern part of Borneo Island (about 25/100 000/year) [1, 2]. Despite the fact that the incidence and mortality of NPC are decreasing as a result of advances in neoadjuvant chemoradiotherapy, patients’ 5-year survival rates remain poor [3, 4]. In the past few decades, NPC patients are almost resistant to common traditional drug treatments in the advanced stages [5]. Therefore, more effective therapeutic agents are urgently needed in the next decades of NPC research.

The antipyretic, analgesic, and anti-inflammatory are the properties of 2,5-dimethyl celecoxib (DMC), a structural counterpart of celecoxib that cannot occupy cyclooxygenase-2 (COX-2) [6]. It also has a number of characteristics that make it a good candidate for repurposing as an anti-cancer treatment in the same way as celecoxib is. In developing malignancies, DMC has been linked in glioblastoma, prostate, leukemia, Burkitt’s lymphoma and pancreatic cancer cells, anti-cancer effects [7–9]. Of note, DMC would not influence the intracellular prostaglandin E2 (PGE2) level, avoid the toxicity profile of celecoxib, which includes gastric ulcers, elevated stroke risks, and cardiovascular thrombotic events [10]. As a result, DMC’s COX-2-independent action could be beneficial for future promising alternative cancer therapies without the conventional adverse effects. However, whether DMC also exhibits anti-tumor effect against NPC is not yet investigated. We investigated the effect of DMC on NPC *in vitro* and *in vivo*, as well as the underlying molecular mechanism that underpins it.

In the past decades, DMC has been reported to possess multiple pharmacological activities, such as aggravated endoplasmic reticulum stress (ER stress), anti-angiogenisis, induction of apoptosis and inhibition of cell cycle progression, which are COX-2-independently involved in the antitumor effects [8, 11–13]. However, the role of DMC on autophagy is not yet investigated. Lysosomal breakdown and the recycling of destroyed cellular components and energy in order to maintain homeostasis is a proteolytic process, known as autophagy, or type II PCD [14]. Autophagic dysfunction, on the other hand, has been linked to a variety of illnesses, including immunological, metabolic, and neurological diseases, including cancer [15]. Many investigations have found that autophagy regulation is a key mechanism linked to anti-cancer therapies [16, 17]. In response to stress signals, such as dietary deficit, cytokine and growth factor reductions, and chemotherapeutic medicines, the functional link between apoptosis and autophagy is complex [18]. For NPC therapy, the development of novel targeted medicines, research in the impacts and basic molecular mechanisms of DMC on autophagy, as well as the apoptosis–autophagy interaction, is necessary.

We found in our study that in NPC cells, DMC had anti-cancer properties both *in vitro* and *in vivo*. We also studied the molecular mechanism by which DMC caused apoptosis and autophagic cell death, which was controlled by the ROS/JNK signaling axis.

## MATERIALS AND METHODS

### Animals

At the Research Animal Centre in Xi’an Jiaotong University (Xi’an, China), BALB/c nude mice, four weeks old were purchased and kept under pathogen-free conditions. In 100 μL PBS, a total of 2.0 × 10^6^ CNE-2 cells were resuspended and intravenously injected into BALB/c nude mice aged six weeks at their right trunks. The mice were divided into two groups (each group *n* = 4), once the tumours attained a size of 4–6 mm. For 16 days, the mice were given vegetable oil and 40 mg/kg DMC orally each day. The average tumour volume was estimated using the equation, volume = (L × W^2^)/2, and the tumour size and body weight were assessed. Experimental mice were immediately euthanized, and the tumours were resected, weighed and photographed, at the end of the treatment. The study was performed in accordance with the First Affiliated Hospital of Xi’an Jiaotong University’s Institutional Animal Care and Use Committee.

### Cells culture and drugs treatment

The Research Centre of Carcinogenesis and Targeted Therapy, Xiangya Hospital, Central South University generously provided the human NPC cell lines, which were CNE-2 and radioresistant CNE-2R. DMC (D7196-5MG) was bought from Sigma-Aldrich and dissolved at 50 mM in 100 per cent DMSO, which was kept at 4°C before being diluted with 1640 medium to different concentrations. And DMC was dissolved at 100 mg/mL in vegetable oil before use *in vivo*. 3-Methyladenine (3-MA, S2767), Hydroxychloroquine Sulphate (CQ, S4430), and SP600125 (#1460) were the reagents of Selleck Chemicals (USA). 3-MA and CQ were dissolved at 50 mM in ddH_2_O, and SP600125 was dissolved at 50 mM in 100% DMSO. MedChem Express delivered N-acetylcysteine (NAC, HY-B0215). Before use in *in-vitro* experiments, these compounds were kept at –20°C and diluted with 1640 medium to the appropriate concentration (final DMSO concentration <0.1 per cent).

### Cells viability assay

The appropriate density of CNE-2 and CNE-2R cell lines were planted into 48-well culture plates, and were cultured overnight at 37°C. DMC was applied to the cells at various concentrations for 24-72 hours. The control group received 0.1 per cent DMSO as a treatment. After treatment, the cells were treated for 2 hours at 37°C with 25 μL of 0.5 mg/mL MTT. After removing the medium, each well was filled with 360 μL of MTT solvent (DMSO) and the plate was shaken for 10 minutes. A microplate reader (PerkinElmer, Waltham, MA, USA) was used to measured the optical densities (ODs) at 490 nm.

### Colony formation assay

The density of 400 and 200 CNE-2 and CNE-2R cells/well were implanted in six-well culture plates overnight, respectively, to study the growth changes of single cells to create a colony. The plates in DMC-containing medium were incubated for 12 days at 37°C. The colonies were maintained for 15 minutes in 4 per cent formaldehyde and then stained for 15 minutes in 0.1 per cent crystal violet in methanol. The cell colonies were finally counted and evaluated. Each experiment was carried out three times in total.

### Apoptosis assay

CNE-2 and CNE-2R cells into six-well culture plates were implanted. When reached 50%–60% concentration, the cells were treated with DMC alone or co-incubation with NAC or SP600125 for the time period indicated. The cells, after treatment, were stained using an Annexin V-PE Apoptosis Detection Kit (BD Biosciences; USA) as directed by the manufacturer. The cells were analysed using flow cytometry (BD FACS Calibur) and the Flowjo software package.

### Transmission electron microscopy

CNE-2 and CNE-2R cells into 10-cm culture dishes were seeded. When reached 50%–60% confluence, the cells were exposed to 0.1% DMSO or 20 μM DMC for 24 hours. The cells were then harvested, centrifuged, and the aqueous phase was discarded, after treatment. The cells for 4 hours at 4°C in sodium phosphate buffer (2 percent glutaraldehyde and paraformaldehyde, each) were then preserved and postfixed for 1.5 hours in 1 per cent osmium tetroxide, water rinsed, and stained for 1 hours in 3 per cent aqueous uranyl acetate. After that, the samples were gradually dehydrated in 50%, 75%, 95%, or 100% alcohol before being embedded in Epon-Araldite resin. Finally, a JEM-1230 transmission electron microscope (JEOL Co., Ltd., Japan) was used to examine these materials.

### Western blotting

The CNE-2 and CNE-2R cells, after treatment, were extracted with the buffer radioimmuno- precipitation assay (RIPA), supplemented with protease and phosphatase inhibitors (Roche, NJ, USA). To determine the protein concentrations, we used the Bradford Protein Assay Kit. The proteins were then separated using SDS-PAGE at 12% or 15% and transferred to PVDF membranes. After blocked, these membranes were incubated overnight at 4°C with the corresponding primary antibodies. Cell Signaling Technology provided anti-LC3 (#4108), P62 (#88588), caspase-3 (#9662), cleaved caspase-3 (#9664), Survivin (#2808), JNK (#9252) and phospho-JNK (#4668). Cleaved PARP (#P09874) was purchased from Abways Technology. Bcl2 (sc-130308) and Bax (sc-6236) were purchased from Santa Cruz Biotechnology. GADPH and β-actin primary antibodies were obtained from Proteintech, China. The bands of protein were developed with a chemiluminescence reagent (Cell Signaling).

### Immunofluorescence

CNE-2 and CNE-2R cells were seeded into culture plates with chamber slides. After treatment, the cells were fixed with 4 per cent formaldehyde and then permeabilized with 0.2 per cent Triton X-100 for 10 minutes. After blocked with 5 per cent BSA in PBST for 1 hours at room temperature, the chamber slides incubated with anti-LC3 antibodies (1:100). At room temperature, the cells were incubated for 1 hours with an appropriate fluorescently conjugated secondary antibody (Invitrogen #A11008; USA) (1:200). With 5 μg/mL DAPI nuclei were treated, followed by imaging of different fields (>5 regions) using a confocal microscope (Leica TCS SP5II, Germany).

### Immunohistochemistry

The tumours promptly preserved in 10% neutralized formaldehyde for 24 hours and then embedded in paraffin after all mice were euthanized. Anti-LC3II primary antibodies (1:50 dilution) were applied overnight at 4°C, followed by 60 minutes at room temperature with a second antibody. A Leica SCN400 slide scanner was used to take the photos.

### Adenovirus infection

CNE-2 and CNE-2R cells on 6-cm culture dishes were plated. When reached 50%–60% confluence, cells were transfected with mRFP-GFP-LC3 lentivirus (Shanghai Genechem Co, Ltd) as directed by the manufacturer. After 48 h, the infected cells were implanted into 24-well culture plates with chamber slides. After treatment for the time period indicated, the expression of green and red fluorescent proteins (GFP and RFP) were detected using a confocal microscope.

### Measurement of intracellular ROS level

In six-well culture plates, CNE-2 and CNE-2R cells were planted. They were treated with 0.1 per cent DMSO or 20 μM, 40 μM DMC for 24 hours once had attained 50–60 per cent proliferation. The cells were examined using a ROS Detection Kit (KeyGEN Biotechnology, China) after treatment. Briefly, the cells were stained with 0.1 mM dichloro-dihydro-fluorescein diacetate (DCFH-DA), after washed three times with serum-free medium for 30 minutes at 37°C. They were then harvested and washed three times. Flow cytometry (BD FACSCalibur) was used finally, to examine the cells after they were resuspended in 400 uL PBS.

### Statistical analysis

Each procedure was carried out three times in total. The data is recorded as a mean ± SD. A comparison between the two groups was performed using the unpaired Student’s *t*-test. The analysis of two-way variance (ANOVA) was used to compare several groups. GraphPad Prism version 6.00 (GraphPad Software, USA) was used for statistical analysis. ^*^P < 0.05, ^**^P < 0.01, and ^***^P < 0.001.

### Availability of data and materials

The data used and analyzed during the current study are available from the corresponding author on reasonable request.

### Ethics approval and consent to participate

All animal experiments were done by protocols approved by the Institutional Animal Care and Use Committee of the First Affiliated Hospital of Xi’an Jiaotong University. The methods were consistent with the approved guidelines.

## RESULTS

### DMC inhibited the NPC proliferation both *in vitro* and *in vivo*

**Figure 1:**
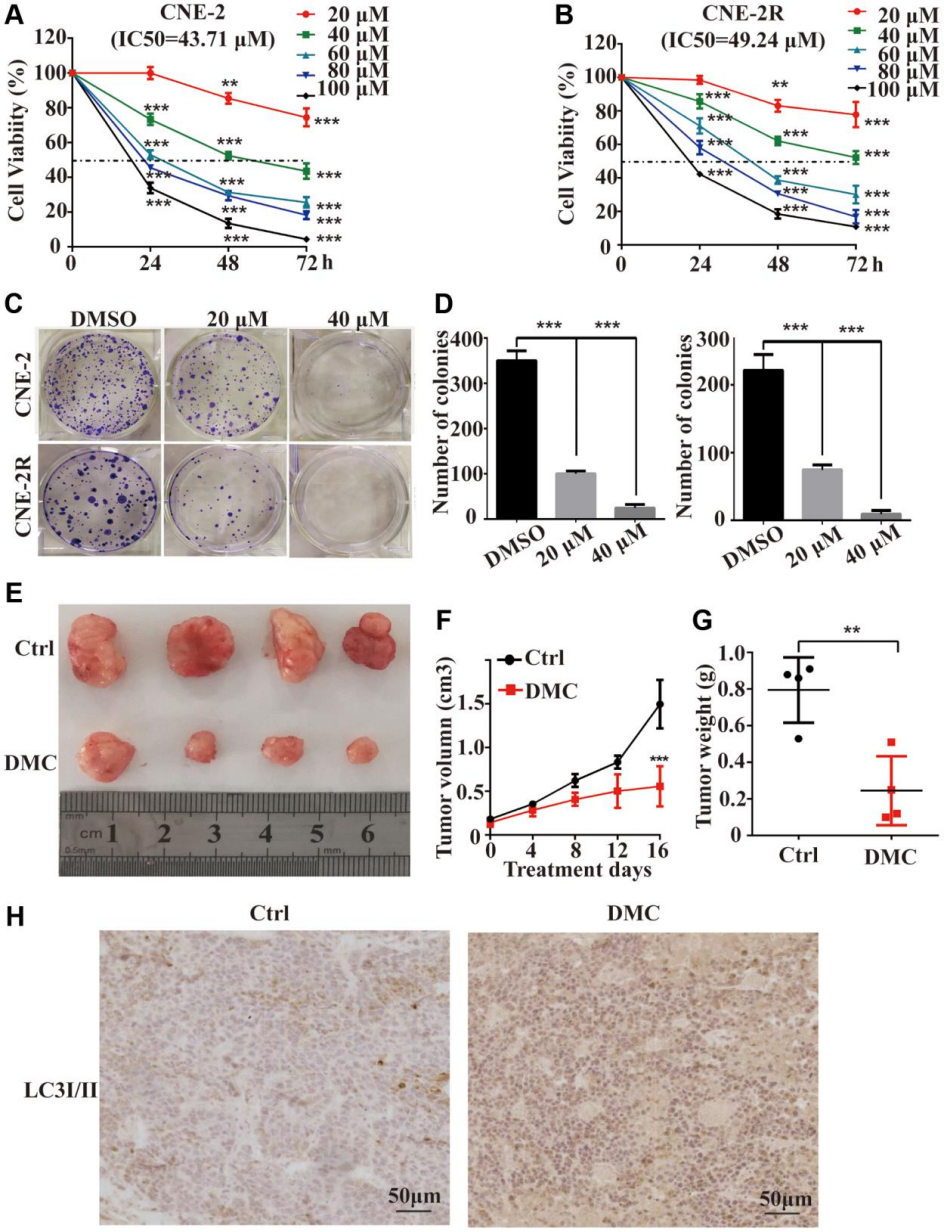
**DMC inhibited the proliferation of NPC both *in vitro* and *in vivo.*** (**A**, **B**) CNE-2 and CNE-2R cells were incubated with 0-100 μM of DMC or 0.1% DMSO for 24 h -72 h, and cells viability was then quantified by the MTT assay. (**C**, **D**) The ability of single CNE-2 and CNE-2R cell to form clones after treating with 0.1% DMSO, 20 μM, or 40 μM DMC assessed by colony formation assay. (**E**) Representative images of BALB/c-nude xenografts from nude mice following the injections of CNE-2 cells in control or DMC group. (**F**) Tumor volumes of xenografts in nude mice for each group was recorded every 4 days after DMC treatment. (**G**) The tumors were removed and the tumor weight for each group was recorded after all mice were killed. (**H**) Tumor specimens were analyzed by immunohistochemical staining with LC3-II antibodies. ^**^P < 0.01, ^**^P < 0.001, and ^***^P < 0.001, significantly different compared with control group.

To examine the anti-proliferative effects of DMC on NPC, CNE-2 and CNE-2R cells with a series of concentrations of DMC (up to 100 μM) were incubated, either for 24 or 48 or 72 hours by using MTT assay. The cell viability decreased in proportion to DMC treatment, revealed by MTT assay in both a dose- and time-dependent manners (Figure 1A and 1B). The IC50 value of DMC in CNE-2 and CNE-2R cells at 48 h were about 43.71 μM and 49.24 μM, respectively. In addition, the cloning formation assay further confirmed the results of MTT assay and showed that DMC inhibited anchorage-dependent growth in both cells (Figure 1C and 1D). For *in vivo* experiment, it showed that DMC remarkably reduced the average tumour volume and weight (Figure 1E, 1F and 1G). Collectively, these data showed DMC exerts the anti-proliferative effect at a safe dose for NPC both *in vitro* and *in vivo*.

### DMC increased the marker proteins of autophagy and autophagosomes in NPC cells

**Figure 2:**
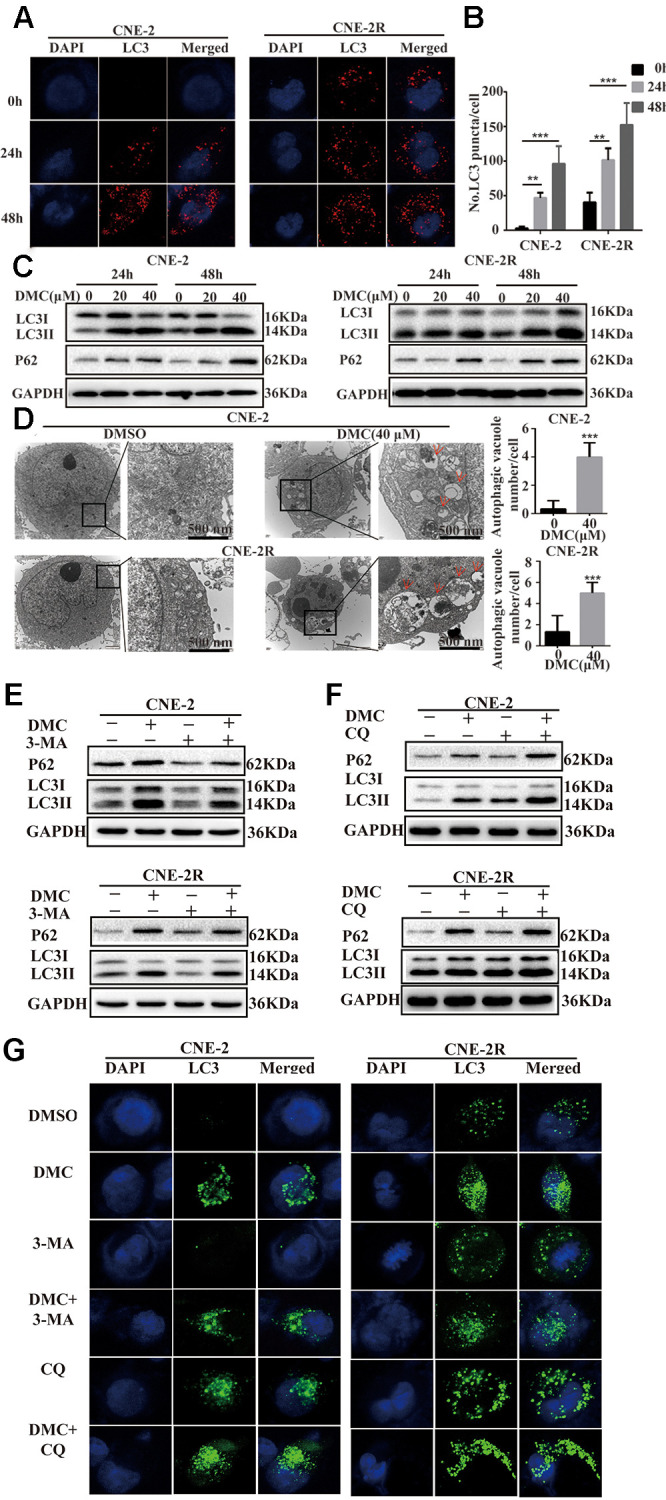
**DMC increased the marker proteins of autophagy and autophagosomes in NPC cells.** (**A**) Representative images of LC3-labelled red fluorescence puncta after 20 μM DMC for 0, 24, 48 h in CNE-2 and CNE-2R cells. Scale bars: 10 μm. (**B**) Statistical analysis histogram of LC3-labeled red fluorescence puncta in per cell and over 30 cells were counted in each group. (*n* = 3; ^**^p < 0.01; ^***^p < 0.005 compared with control group). (**C**) Western blotting analysis of LC3-I, LC3-II and P62 levels in CNE-2 and CNE-2R cells treated with the indicated concentrations of DMC for 24 h and 48 h. GAPDH was used as a loading control. (**D**) Transmission electron microscopy images in CNE-2 and CNE-2R cells after treating with control or DMC (40 μM) for 24 h. Red arrows: autophagosomes. Scale bars: 500 nm. (**E**, **F**) Western blotting analysis of LC3-I, LC3-II and P62 levels in CNE-2 and CNE-2R cells after treating with 0.1% DMSO or 20 μM DMC in the absence or presence of 3-MA (5 mM) and CQ (20 μM) for 24 h. GAPDH was used as a loading control. (**G**) Representative images of LC3-labelled green fluorescence puncta after treating with 0.1% DMSO or 20 μM DMC in the absence or presence of 3-MA (5 mM) and CQ (20 μM) for 24 h in CNE-2 and CNE-2R cells. Scale bars: 10 μm.

To explore the effects of DMC on autophagy. The fluorescent puncta formation of autophagosomes was first discovered by immunofluorescence test in CNE-2 and CNE-2R cells. After being exposed to 20 μM DMC for 0, 24 and 48 hours, the production of LC3-labelled red fluorescence puncta showed a clear rise (Figure 2A and 2B). Then, using a western blotting technique, we examined various autophagy marker proteins in NPC cells following DMC therapy. DMC treatment increased the level of LC3-II and P62, according to the findings (Figure 2C). LC3-II expression was also enhanced following DMC therapy *in vivo*, according to immunohistochemical labelling (Figure 1H). The use of electron microscopy to detect autophagy is known as direct evidence. Transmission electron microscopy (TEM) revealed that in DMC-treated cells, the cytoplasmic double-membrane vesicular structures (shown by red arrows in Figure 2D) were considerably enhanced compared to control cells.

To offer additional evidence of DMC-modulated autophagy, we utilized two autophagy inhibitors: 3-methyladenine (3-MA) and hydroxychloroquine sulphate (CQ). Western blotting data showed that the increased level of LC3-II and P62 induced by DMC were partially abolished under 3-MA treatment (Figure 2E). In contrast, CQ aggravated the protein level of LC3-II and P62 induced by DMC (Figure 2F). Moreover, LC3-labelled green fluorescence was analyzed by immunofluorescence assay together supported the above data (Figure 2G). Thus, our data demonstrated that DMC increased the marker proteins of autophagy and autophagosomes in a dosage- and time-dependent manner for NPC, suggesting DMC may be a novel autophagy inducer in NPC.

### DMC induced autophagy flux in NPC cells

**Figure 3:**
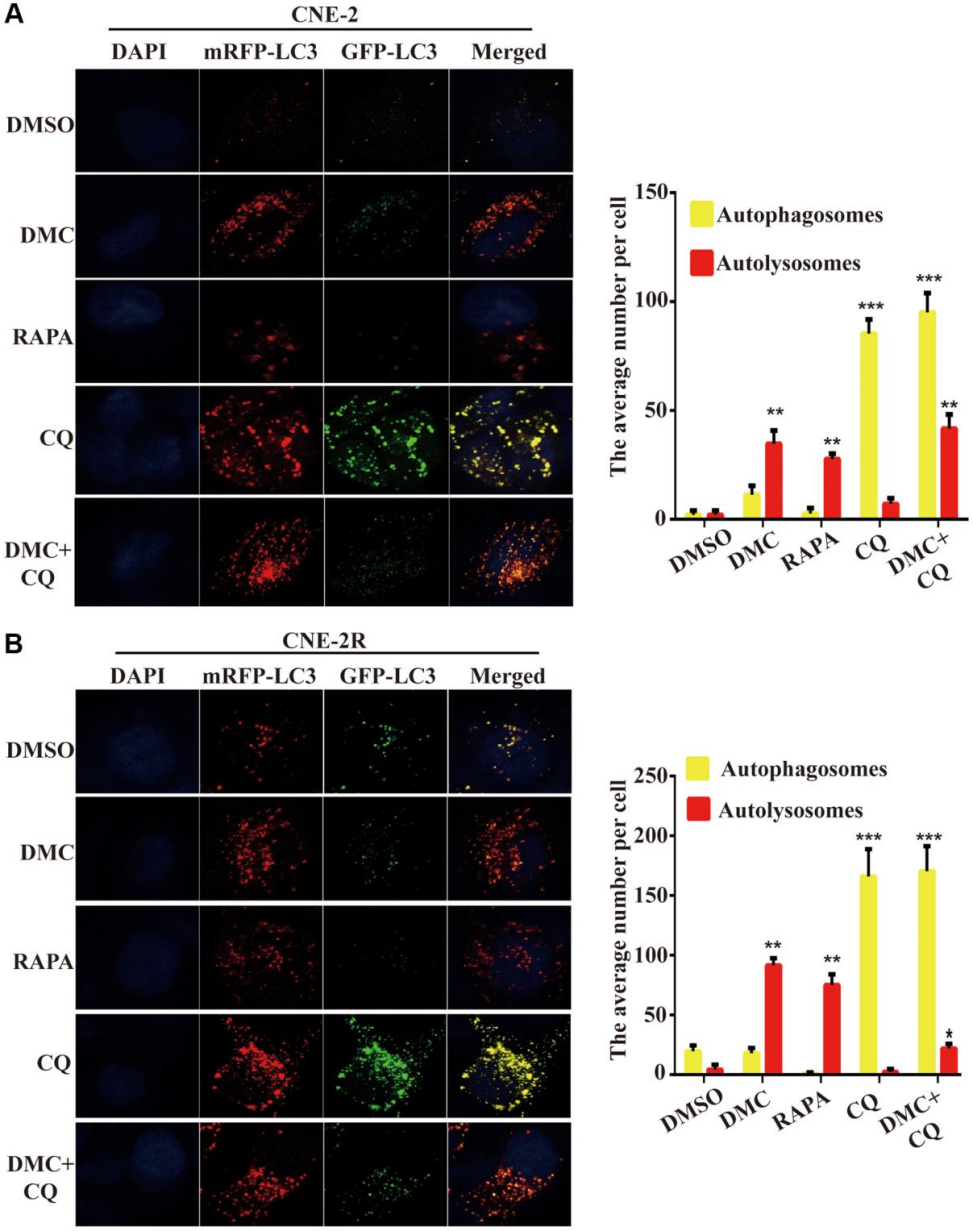
**DMC induced autophagic flux in NPC cells.** (**A**) Fluorescence photographs of CNE-2 and (**B**) CNE-2R cells transfected with mRFP-GFP-LC3B reporter. Cells were treated with 0.1% DMSO, 20 μM DMC, 10 μM RAPA, 20 μM CQ or DMC+CQ for 4 h. CQ-treated cells were used as positive controls. Nuclei were stained with DAPI. Scale bar: 10 μm. Average numbers of autophagosomes (yellow puncta) and autolysosomes (red puncta) per cell, and over 30 cells were counted in each condition. (*n* = 3; ^**^P < 0.01; ^***^P < 0.001 compared with control group).

Since the increased LC3-I to LC3-II conversion and autophagosomes accumulation may be related to either increased formation of autophagosomes by autophagy induction or abnormal degradation of autophagosomes caused by autophagic flux block [19]. Thus, to visualize LC3-labelled cytoplasmic vacuolation dynamically, the mRFP-GFP-LC3 adenovirus was used to transfect cells. The fluorescence of GFP, but not that of RFP, disappears in the acidic environment of autolysosome formation according to the typical changes of autophagic flux that yellow puncta of the overlapping GFP and RFP signals correspond to immature autophagosomes, whereas autolysosomes only emit red fluorescence [20]. In our study, Figure 3A and 3B showed that CQ markedly increased the number of yellow puncta in NPC cells. In contrast, similar to the RAPA-treated group that typical induced autophagic flux, DMC caused many red puncta and several yellow puncta. Moreover, co-incubation of cells with DMC and CQ further led to the decrease of yellow puncta and the increase of red puncta compared to CQ treatment alone. These data confirmed that DMC induced autophagy and autophagy flux in NPC cells.

### DMC-induced autophagy is mediated through activating ROS/JNK axis in NPC cells

**Figure 4:**
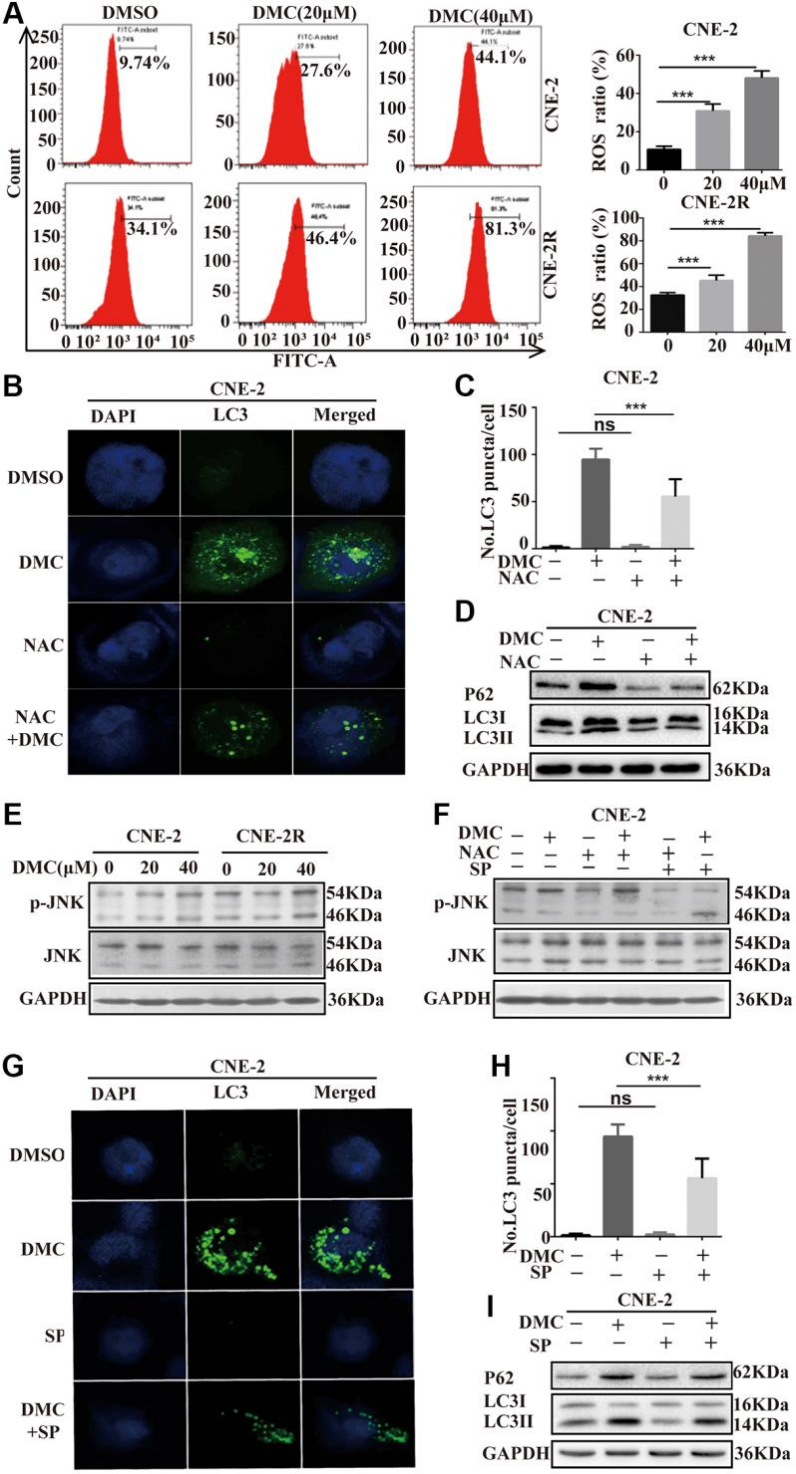
**DMC-induced autophagy is mediated through activating ROS/JNK axis in NPC cells.** (**A**) CNE-2 and CNE-2R cells were treated with 20, 40 μM DMC or 0.1% DMSO for 24 h, and then the ROS level was measured and analyzed by flow cytometry. Statistical analysis histogram of the ROS positive percentage. (**B**, **C**) Representative images of LC3-labelled green fluorescence puncta in CNE-2 cells after NAC (5 mM) pretreatment for 4 h. Scale bar: 10 μm. Nuclei were stained with DAPI. Histogram of LC3-labelled green fluorescence puncta per cell and over 30 cells were counted in each group. (**D**) Western blotting analysis of LC3-I, LC3-II and P62 levels in CNE-2 cells as Figure 4B, GAPDH was used as a loading control. (**E**) Western blotting analysis of of p-JNK and JNK protein expressions in CNE-2 and CNE-2R cells treated with 20, 40 μM DMC or 0.1% DMSO for 24 h. (**F**) Western blotting analysis of of p-JNK and JNK protein expressions in CNE-2 cells treated with 0.1% DMSO or 20 μM DMC for 24 h in the absence or presence of NAC (5 mM) pretreatment for 4 h and SP600125 (30 μM) pretreatment for 1 h. (**G**, **H**) Representative images of LC3-labelled green fluorescence puncta in CNE-2 cells after SP600125 (30 μM) pretreatment for 1 h. Scale bar: 10 μm. Nuclei were stained with DAPI. Histogram of LC3-labelled green fluorescence puncta per cell and over 30 cells were counted in each group. (**I**) Western blotting analysis of LC3-I, LC3-II and P62 protein expressions in CNE-2 cells as Figure 4G. ^**^P < 0.01 and ^***^P < 0.001, significantly different compared with control group.

In order to understand the underlying mechanisms of DMC-mediated autophagy, we analyze the intracellular ROS level following DMC therapy using DCF-DA, a specific ROS-detecting fluorescent dye. As shown in Figure 4A, DMC increased the number of DCF-positive cells in a dose-related manner, indicating DMC significantly induced ROS. The antioxidant N-acetyl cysteine (NAC), which acts as ROS scavenger, was used to investigate further role of ROS in autophagy by DMC. They were pretreated with NAC for 4 hours. Expectedly, LC3-labelled cytoplasmic puncta formation (Figure 4B, 4C) and LC3-II/I level (Figure 4D) inhibited by NAC pretreatment were effectively increased after DMC. Such results suggested DMC-induced autophagy is mediated through intracellular ROS accumulation in NPC cells.

In addition, we focused on the involvement of JNK signalling, one of the primary stress-responsive signalling pathways in cells with a button controlled by ROS [21], to see if the mechanisms of ROS-mediated autophagy could be identified. DMC enhanced JNK signaling, as demonstrated by a significant increase in p-JNK, but total JNK remained unchanged (Figure 4E). The decrease of p-JNK after pretreatment with NAC suggests that ROS are involved in DMC-mediated JNK signaling activation (Figure 4F). Moreover, we utilized the specific JNK inhibitor, SP600125 and found it effectively suppressed DMC-induced autophagy in CNE-2 cells, evidenced by (i) reduced LC3-labelled cytoplasmic puncta formation (Figure 4G, 4H) and (ii) reduced LC3-II/I level (Figure 4I). Therefore, all above data indicated that DMC induced autophagy by activating the ROS/JNK signaling.

### DMC induced apoptosis and autophagic death in NPC cells

**Figure 5:**
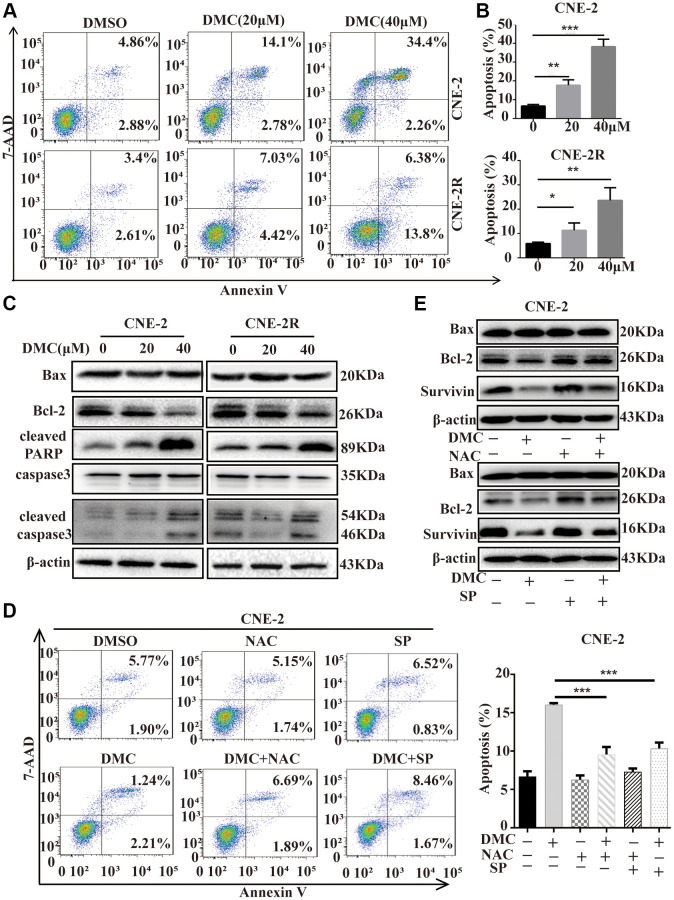
**DMC induced apoptosis and autophagic death in NPC cells.** (**A**) CNE-2 and CNE-2R cells were treated with 0.1% DMSO or DMC (20 and 40 μM) for 48 h, and then apoptotic cells were detected with the annexin V-PE/7-AAD kit and analysed by flow cytometry. (**B**) Statistical analysis histogram of apoptotic cells ratio. (**C**) Cells were treated with DMC 0.1% DMSO or DMC (20 and 40 μM) for 48 h, and the expression levels of apoptosis-related proteins (cleaved-PARP, cleaved-caspase-3, caspase-3, Bax, and Bcl-2) were tested in cells by Western blotting. β-actin was used as a normalization control. (**D**) CNE-2 cells were treated with 0.1% DMSO or 20 μM DMC for 24 h in the absence or presence of NAC (5 mM) pretreatment for 1h or SP600125 (30 μM) pretreatment for 1 h, and then apoptotic cells were detected by flow cytometry. Statistical analysis histogram of apoptotic cells ratio. (**E**) The expression levels of Bax, Bcl-2 and Survivin were tested in CNE-2 cells as Figure 5D by Western blotting. ^**^P < 0.01 and ^***^P < 0.001, significantly different compared with control group.

A lot of studies have been taken in determining the perspective roles of autophagy in cancer, which represent the impacts of chemoradiotherapy on avoiding or inducing apoptotic cell death [22]. Therefore, we need to comprehend the relationship between DMC-induced autophagy and apoptosis in NPC cells. Firstly, we exploited flow cytometry determining the effects of DMC on apoptosis showing that apoptosis cells were augmented after DMC treatment (Figure 5A and 5B). Moreover, Bcl-2/Bax and Survivin expression were significantly decreased, whereas the levels of cleaved-PARP and cleaved caspase-3 were increased after DMC treatment (Figure 5C). Together, these data confirmed that DMC induced apoptosis in NPC cells. Subsequently, we used NAC and SP600125 to inhibit DMC-induced autophagy in CNE-2 cells, in order to investigate whether inhibition of autophagy promotes cells from apoptosis by flow cytometry and western blotting assay. As was showed in Figure 5D and 5E, pretreatment with NAC and SP600125 decreased DMC-upregulated apoptotic ratio as well as Bcl-2/Bax and Survivin expression after DMC treatment in CNE-2 cells. All the above-mentioned data indicated that DMC induced apoptosis in NPC cells and the autophagy induced by DMC may be pro-apoptotic.

## DISCUSSION

Concurrent cisplatin or 5-fluorouracil (5-FU)-based chemoradiotherapy is regarded as the standard of treatment for locoregionally advanced NPC [23]. However, NPC patients are almost resistant to common traditional drug treatments in the advanced stages. DMC dramatically decreased NPC cell proliferation and tumor formation in a xenograft model in this study, with no usual harmful side effects. Furthermore, we investigated that DMC induced apoptosis and autophagic cell death are regulated by the ROS/JNK signaling. Based on these findings, it was suggested that DMC, as an autophagy inducer, may be an effective and reliable antitumor drug for NPC.

In the past decades, DMC has been reported to induce apoptosis involved in the antitumor effects [8]. Consistently, DMC also induces apoptosis in NPC cells, proved by an increase of the cleavage of caspase-3 and PARP as well as a decrease in Bcl-2/Bax level after DMC exposure. In addition, a considerable amount of literature has demonstrated that anti-cancer drugs caused cells death is found to be associated with autophagy [24, 25]. Of note, recent studies demonstrated celecoxib is determined to be autophagy inducer or inhibitor linked with preventing the occurrence and development of carcinoma [26, 27]. However, the effect on autophagy of DMC in NPC has not been well established. In this study, DMC was found to induce autophagy via increasing LC3-II expression and the production of autophagosomes, in NPC cells. The conjugation of cytosolic LC3-I to phosphatidyl ethanolamine on the surface of autophagosomes produces LC3-II, a typical marker for autophagosomes [28]. Therefore, the level of LC3-II and the LC-3 puncta formation have been widely used as reliable methods for autophagy monitoring. Autophagy is a dynamic and multi-step process that includes the production of autophagosomes, autolysosome, and the final degradation of autophagic cargo. The overall catabolic process of autophagy is referred to as autophagic flux [29]. In this study, we further observed DMC activated autophagic flux in a tandem mRFP-GFP-LC3 dual fluorescence system. Taken together, these results indicated that DMC induces apoptosis and completely autophagy in NPC cells.

Numerous investigations have previously reported that ROS produced by mitochondria are the primary stimulators of autophagy in the presence of oxidative stress [30, 31]. In hepatocellular carcinoma, a novel celecoxib derivative, OSU-03012 effectively induces ROS-mediated autophagy, according to Gao and colleagues [32]. Our current findings show that DMC induced a significant increase in ROS production, whereas the effect of DMC on autophagy was dramatically reversed when treated with NAC, a ROS scavenger. According to the previous research, in the activation of MAPK pathways, the intracellular ROS accumulation plays a major role, that can impair mitochondrial function [33]. The JNK, p38, and ERK are mainly included in the MAP-kinase pathway. The pathway is involved in cell proliferation, differentiation, stress adaptation, and apoptosis [34]. OSU-03012 also suppressed c-Jun NH2-terminal kinase/signal transducers and transcription activators, and MAPK pathways in multiple myeloma cells, according to Zhang and colleagues [35]. Whether there exists an association between ROS/JNK axis and DMC-induced autophagy, remains unclear. We observed in this study, that DMC therapy resulted in a substantial increase in JNK phosphorylation in a dose-depended way. The pre-treatment with SP600125 revealed that JNK singling activation had a significant role in the control of the DMC-induced autophagy. We also discovered that pretreatment with NAC reduced the phosphorylation of JNK caused by DMC. These findings demonstrated that DMC could trigger autophagy by activating the ROS/JNK axis.

Many anti-cancer treatments have been shown to activate autophagy, and the the effects of autophagy can be classified as pro-death or pro-survival functions in response to stress induced by chemotherapeutic drugs [36, 37]. Alkannin promotes ROS-mediated mitochondrial malfunction and activation of the JNK pathway, according to Zheng, which causes cytotoxic autophagy and apoptosis [38]. And recent study suggested that Metformin induces cell cycle arrest, apoptosis and autophagic death through ROS/JNK signaling pathway in human osteosarcoma [39]. In some cases, however, autophagy contributes to chemotherapy resistance via a cytoprotective mechanism in cancer cells. The c-Jun N-terminal kinase-mediated pathway, for example, inhibits apoptosis of human prostate cancer cells treated with celecoxib [40]. There have been conflicting reports about celecoxib’s effect on autophagy, indicating that whether celecoxib produces autophagic cells death or whether autophagy suppression increases celecoxib-induced apoptosis changes depending on the tumor types [41]. Thus, an important question for the use of DMC in NPC treatment is ‘should we try to enhance autophagy or inhibit it’? DMC-induced autophagy partially blocked by SP600125 resulted in a significant decrease of apoptotic cells and apoptosis-related protein in NPC. These findings strongly suggest that autophagy triggered by DMC has a cytotoxic impact on NPC cells, and that combining DMC with autophagy inhibitors may be a sensible NPC therapeutic strategy. However, DMC induces autophagic death via activation of ROS/JNK axis in NPC, which requires further investigation in animal and even clinical trials.

In conclusion, we concluded that DMC might be a potential anti-cancer agent through inducing apoptosis and autophagy in NPC. Moreover, we revealed the mechanism whereby DMC induces autophagy by promoting ROS/JNK axis. Autophagy induced by DMC may have a cytotoxic role, as evidenced by the inhibition of ROS/JNK axis decreased the apoptosis ratio induced by DMC. These findings validated that the potential anti-cancer molecular mechanism of DMC will help to define molecular targets and better treatment therapies for NPC in near future.
